# Fertility Preservation in Patients With Cancer: Insights From a National Survey of Moroccan Oncologists

**DOI:** 10.7759/cureus.104910

**Published:** 2026-03-09

**Authors:** Khadija Darif, Ayoub Mars, Mounia Amzerin, Fatima Zahrae Elmrabet

**Affiliations:** 1 Medical Oncology, Ahmad Bin Zayed Al Nahyan Center of Cancer Treatment, Mohammed VI University Hospital of Tangier, Faculty of Medicine and Pharmacy of Tangier, Abdelmalek Essaadi University, Tangier, MAR

**Keywords:** cancer survivors, fertility preservation, healthcare barriers, oncofertility, oncologists’ awareness and practice

## Abstract

Background

In Morocco, as in many Arab countries, a relatively high proportion of patients with cancer are young adults compared with Western populations. With continuous advances in curative therapies, survival rates and prospects for long-term remission have significantly improved. Consequently, fertility preservation has evolved from a secondary concern into an essential component of comprehensive cancer care.

Objective

This study aims to assess the knowledge, attitudes, and practices of Moroccan medical oncologists and radiotherapists regarding fertility preservation in patients with cancer and to identify the main barriers and unmet needs in this field.

Methods

A descriptive cross-sectional study was conducted using an online questionnaire distributed to oncologists and radiotherapists practicing in Morocco. The survey explored awareness of fertility preservation centers, frequency of discussion with patients, referral patterns, and perceived challenges. A total of 70 physicians participated.

Results

Among respondents, 85% were medical oncologists, mostly working in the public sector. Only 37 physicians were aware of existing fertility preservation centers. Although most recognized the importance of the issue, just 38% systematically discussed fertility preservation with their patients. About half had referred patients to specialized centers, and 24 physicians reported successful preservation in at least one case. Reported barriers included the lack of dedicated structures, financial constraints, and the absence of national guidelines or standardized referral pathways.

Conclusion

This study highlights a significant gap between awareness and practice regarding fertility preservation among Moroccan oncologists. Developing a national oncofertility framework, expanding specialized facilities, and providing targeted education and multidisciplinary collaboration are urgently needed to ensure equitable access to fertility preservation for young patients with cancer in Morocco.

## Introduction

Advances in cancer diagnosis and treatment have significantly improved survival rates among young patients worldwide. As a result, the long-term quality of life, including reproductive health, has become a major concern in oncology care. Fertility preservation is now recognized as a critical component of cancer management, recommended by international guidelines such as those of the American Society of Clinical Oncology (ASCO) and the European Society for Medical Oncology (ESMO), which emphasize early counseling and referral before the initiation of gonadotoxic therapy [1-3].

In low- and middle-income countries (LMICs), and particularly in the Middle East and North Africa (MENA) region, the integration of fertility preservation into oncology practice remains limited. Several studies have shown that despite growing awareness among healthcare providers, barriers such as the lack of specialized centers, limited financial resources, and the absence of clear national guidelines continue to hinder access to fertility-preserving options [4-6]. Furthermore, cultural and social perceptions around fertility and cancer may influence both physician-patient communication and patients’ willingness to pursue preservation strategies [4-6].

In Morocco, as in several Arab countries, the age distribution of patients with cancer is shifted toward younger populations compared with Western countries [7]. With the steady improvement of diagnostic and therapeutic capacities, the number of patients achieving prolonged remission or cure is increasing, bringing fertility preservation to the forefront of survivorship care. However, to date, fertility preservation remains a poorly explored domain in Moroccan oncology practice. No national guidelines or structured referral pathways exist, and information for both physicians and patients is often limited.

In this context, understanding healthcare providers’ perceptions and practices is essential to identify existing gaps and guide future strategies.

This study aimed to evaluate the knowledge, attitudes, and practices of Moroccan medical oncologists and radiotherapists regarding fertility preservation in patients with cancer and to highlight the main barriers to its implementation.

## Materials and methods

Study population and participants

This national, cross-sectional survey targeted medical and radiation oncologists practicing in Morocco. Eligible participants included physicians currently involved in the management of adult or pediatric patients with cancer in either public or private healthcare institutions. Inclusion criteria were as follows: (1) practicing as a certified medical or radiation oncologist in Morocco at the time of the survey and (2) direct involvement in patient care. Exclusion criteria included residents or trainees not yet certified, retired oncologists no longer in clinical practice, and incomplete questionnaire responses.

Participants were identified and invited through national professional oncology networks and scientific societies. The survey was open to all eligible oncologists nationwide without restriction on age, years of experience, or institution type, ensuring broad representation of current clinical practice.

Study design and data collection

A structured, web-based questionnaire consisting of 21 items was developed to evaluate oncologists’ knowledge, attitudes, and practices relating to fertility preservation in patients with cancer. The questionnaire included four domains: demographic and professional characteristics (age group, gender, specialty, sector of practice, years of experience, and region of practice), knowledge of fertility preservation (awareness of available techniques, existing centers, and international guidelines), clinical practice patterns (frequency of fertility counseling, referral practices, and patient selection criteria), and perceived barriers and needs (organizational, legal, financial, cultural, and clinical constraints).

The questionnaire was created using Google Forms (Google, Inc., Mountain View, CA) and disseminated electronically through oncology mailing lists, professional societies, and institutional networks. Before accessing the survey, participants were informed about the study objectives, anonymity, and voluntary participation; electronic consent was obtained prior to questionnaire initiation. No identifying data were collected. The survey remained open for approximately one month.

Sample size considerations

As this was an exploratory, descriptive national survey, no formal sample size calculation was performed. All eligible oncologists reached through national networks were invited to participate using a convenience sampling approach. A total of 70 respondents completed the questionnaire and were included in the final analysis.

Study parameters and outcomes

The primary outcome was to assess the proportion of oncologists who routinely discuss fertility preservation with eligible patients with cancer.

Secondary outcomes included awareness of available fertility preservation techniques and centers, familiarity with international guidelines, referral patterns, perceived barriers to implementation, interest in training, or institutional protocol development.

Data management and statistical analysis

Survey responses were recorded automatically in Google Forms and exported to Microsoft Excel (Microsoft Corp., Redmond, WA) for analysis. Data quality was checked for completeness, and only fully completed responses were retained. Descriptive statistics were used to summarize the data. Categorical variables were presented as frequencies and percentages, while continuous variables (where applicable) were summarized as means or medians. No inferential statistical analyses were conducted due to the descriptive and exploratory nature of the study.

Participation was voluntary and anonymous, and no financial or institutional incentives were provided.

Sample size calculation/power analysis

The present study was designed as an exploratory, descriptive national survey. Therefore, no formal a priori sample size calculation or power analysis was performed. Our objective was to obtain the broadest possible representation of medical and radiation oncologists currently practicing in Morocco rather than to test a specific hypothesis or detect a predefined statistical effect size. All eligible oncologists who could be reached through national professional networks were invited to participate, and the final sample consisted of 70 respondents. This pragmatic approach to sampling is consistent with previous surveys assessing knowledge and attitudes toward fertility preservation among oncologists in comparable healthcare settings, where sample sizes typically ranged between 50 and 150 participants. Given the descriptive nature of our analyses, which were limited to frequency and percentage distributions, inferential hypothesis testing was not performed, and a formal power analysis was therefore not required.

## Results

A total of 70 oncologists completed the questionnaire. The median age was 30 years (range: 26-45), with a predominance of female participants (n=52, 74%). Most respondents were medical oncologists (n=60, 86%), followed by radiation oncologists (n=10, 14%), and nearly all worked in the public sector (n=66, 94%), mainly within university or regional oncology centers. Regarding professional experience, the majority (n=52, 74%) had been practicing for less than five years, while 13 (19%) had 5-10 years of experience, and five (7%) had more than 10 years of practice, reflecting the predominance of young oncologists in Moroccan oncology practice (Table 1).

**Table 1:** Characteristics of the respondents (N=70)

Variable	Category	Number	%
Age (years)	Median (range)	30 (26-45)	-
Sex	Female/male	52/18	74/26
Specialty	Medical oncology/radiation oncology	60/10	86/14
Main practice setting	Public sector/private sector	66/4	94/6
Years of practice	<5 years/5-10 years/>10 years	52/13/5	74/19/7

All participants reported awareness of fertility preservation (n=70, 100%). However, familiarity with international guidelines (ASCO, ESMO, and European Society of Human Reproduction and Embryology (ESHRE)) was limited: 22 (31%) were fully aware, 25 (36%) had partial knowledge, and 23 (33%) were unaware. Awareness of fertility preservation methods differed by gender: all participants were aware of sperm cryopreservation for men, while for female patients, 95% knew about oocyte cryopreservation, 40% about embryo cryopreservation, 30% about ovarian tissue cryopreservation, and 55% about ovarian suppression with LH-RH analogues. Several participants reported awareness of multiple strategies, most commonly oocyte cryopreservation combined with LH-RH analogue suppression.

Regarding clinical practice, only 37% of the respondents stated that they always discuss fertility preservation with young patients prior to initiating potentially gonadotoxic treatment, 21% reported often discussing it, 40% rarely, and 1.4% never. Awareness of existing fertility preservation centers in Morocco was moderate, with 44 (63%) participants indicating knowledge of such facilities (Table 2).

**Table 2:** Knowledge and awareness regarding fertility preservation (N=70) ASCO: American Society of Clinical Oncology, ESMO: European Society for Medical Oncology, ESHRE: European Society of Human Reproduction and Embryology

Variable	Category	Number	%
Awareness of fertility preservation	Yes	70	100
Knowledge of international fertility preservation guidelines (ASCO, ESMO, and ESHRE)	Yes	22	31
	Partially	25	36
	No	23	33
Awareness of fertility preservation centers in Morocco	Yes	44	63
	No	26	37
Perception of Moroccan law adequacy on fertility preservation	Yes	1	2
	No	28	40
	Unsure	41	58
Referral of patients to fertility preservation centers	Yes	33	47
	No	37	53

Concerning the legal framework, 41 (58%) were uncertain about the adequacy of Moroccan legislation regulating fertility preservation, 28 (40%) considered it insufficient, and only one participant (2%) considered it adequate. Overall, 33 (47%) reported having referred at least one patient to a fertility preservation center. Among those who had not referred patients (n=37, 53%), the main barriers cited were lack of information about available centers (33%), high costs for patients (37%), and time constraints or therapeutic urgency (1%). Most respondents (n=56, 80%) believed that fertility preservation should be systematically proposed to all young patients, whereas 19% thought it should depend on the clinical situation, and only one participant (1%) disagreed.

Participants expressed strong interest in initiatives to improve fertility preservation practices: 66 (94.3%) were interested in receiving specific training, 62 (88.6%) supported the implementation of standardized departmental protocols, and 60 (85.7%) favored establishing collaborations with reproductive medicine centers.

## Discussion

Fertility preservation has become an essential component of supportive care for young patients with cancer worldwide. As advances in cancer therapy continue to improve survival outcomes, increasing attention is being paid to survivorship issues, including reproductive potential and long-term quality of life. In our national survey of 70 medical oncologists and radiotherapists in Morocco, we found that although awareness of fertility preservation techniques was nearly universal, their systematic integration into daily practice remains limited. Only 38% of respondents reported routinely discussing fertility preservation with eligible patients, and 47% had referred at least one patient to a specialized center. This discrepancy between awareness and implementation has also been described in previous studies and reflects the difficulty of translating theoretical knowledge into routine clinical practice.

The ASCO guidelines clearly recommend that fertility risks and preservation options be discussed with all patients of reproductive age prior to initiating potentially gonadotoxic therapy [8]. Similarly, ESMO and ESGO emphasize the need for a multidisciplinary approach involving oncologists, reproductive specialists, and psychologists in order to ensure individualized, patient-centered care [9,10]. However, as demonstrated in our study, the application of these recommendations in Morocco is still incomplete. This situation parallels findings from other regions, where awareness is generally high among oncologists, yet fertility preservation counseling is not systematically implemented in daily practice [4,5].

The barriers reported by our respondents, including absence of dedicated centers, high costs, uncertainty about the legal framework, and therapeutic urgency, are consistent with observations from other Arab countries and low- and middle-income healthcare settings [4,5]. These structural and contextual obstacles appear to play a major role in limiting access to fertility preservation. Importantly, similar challenges have also been reported in high-income countries such as Canada, where referral rates and access depend strongly on legislation, insurance coverage, and institutional policy rather than knowledge alone [11]. This suggests that the issue is not only economic, but also organizational and regulatory.

Knowledge of international guidelines was limited among Moroccan oncologists, with only 31% reporting familiarity with key recommendations, while 36% had partial awareness and 33% were unaware. Comparable results have been reported in other countries, highlighting the need for continuing medical education and better dissemination of standardized protocols [4,5]. Encouragingly, most participants in our study expressed strong interest in improvement strategies such as specialized training (94%), introduction of departmental protocols (88.6%), and establishment of formal collaborations with reproductive medicine centers (85.7%), indicating a high level of readiness for change.

Legal clarity and institutional support also emerged as critical determinants of practice. More than half of the participants were uncertain about the adequacy of Moroccan legislation, and 40% considered it insufficient to support fertility preservation. This uncertainty may contribute to hesitancy in initiating discussions or referrals. Development of clear national guidelines and ethical frameworks would therefore be essential to ensure equitable and standardized access across the country.

The creation of specialized oncofertility units within oncology centers, as implemented in several healthcare systems internationally, could facilitate earlier counseling, reduce delays between diagnosis and fertility preservation procedures, and strengthen multidisciplinary coordination [8-10]. Integrating fertility preservation into the public health system and expanding financial coverage for cryopreservation would further improve accessibility. In addition, culturally adapted patient education initiatives in Morocco could increase awareness and acceptance of fertility preservation options among young patients with cancer and their families.

Overall, our findings suggest that Moroccan oncologists recognize the importance of systematically integrating fertility preservation into cancer care for young patients. However, significant structural, regulatory, and educational gaps persist. Addressing these barriers through training, national protocols, financial support mechanisms, institutional development, and a clear legal framework would help align current practice with international standards and improve survivorship outcomes.

## Conclusions

Our study clearly shows that, in Morocco, oncologists are aware of fertility preservation, but translating this awareness into routine practice is still limited. Only a minority of physicians discuss fertility preservation systematically with their patients or refer them to specialized centers. The main obstacles include the lack of dedicated facilities, financial constraints, unclear legal guidance, and the urgency of starting cancer treatment. Interestingly, similar challenges exist even in high-income countries, such as Canada, which shows that these barriers are not only related to resources but also to organization and policy.

These findings highlight the urgent need to develop a national oncofertility strategy in Morocco. This should include standardized guidelines, specialized oncofertility units, targeted training for healthcare professionals, and measures to reduce the financial burden for patients. Raising awareness among patients and fostering close collaboration between oncology and reproductive medicine teams are also essential. By taking these steps, Morocco can bridge the gap between knowledge and practice, ensuring that fertility preservation becomes a routine, equitable part of cancer care for young patients. This would align national practice with international recommendations and, most importantly, improve the long-term quality of life for cancer survivors.

## Appendices

Table 3 presents the questionnaire used in the present study. 

**Table 3:** Questionnaire ASCO: American Society of Clinical Oncology, ESMO: European Society for Medical Oncology, ESHRE: European Society of Human Reproduction and Embryology

Section	#	Question	Response Options
I. Sociodemographic and Professional Data	1	Age	_____
	2	Sex	☐ Male ☐ Female
	3	Specialty	☐ Medical oncology ☐ Radiation oncology ☐ Other: _____
	4	Years of practice	☐ <5 years ☐ 5-10 years ☐ >10 years
	5	Main practice setting	☐ Public sector ☐ Private sector ☐ Mixed
	6	Region	_____
II. Knowledge	7	Have you heard of fertility preservation?	☐ Yes ☐ No
	8	Are you familiar with international guidelines (ASCO, ESMO, and ESHRE)?	☐ Yes ☐ No ☐ Partially
	9	Which fertility preservation methods are you aware of (multiple answers)?	☐ Sperm cryopreservation ☐ Oocyte cryopreservation ☐ Embryo cryopreservation ☐ Ovarian tissue cryopreservation ☐ Ovarian suppression with LH-RH analogues ☐ Other: _____
	10	Are you aware of any centers in Morocco offering these techniques?	☐ Yes ☐ No If yes, please specify: _____
III. Attitudes	11	Should fertility preservation be systematically offered to young patients with cancer?	☐ Yes ☐ No ☐ Depending on the case
	12	In your opinion, what are the main obstacles in Morocco (multiple answers)?	☐ Lack of physician awareness ☐ High cost/lack of insurance coverage ☐ Absence of clear legal framework ☐ Constraints due to urgency of treatment ☐ Sociocultural barriers ☐ Other: _____
	13	Do you think the current Moroccan law adequately regulates fertility preservation?	☐ Yes ☐ No ☐ Don’t know
IV. Practices	14	Do you discuss fertility with young patients before gonadotoxic treatment?	☐ Always ☐ Often ☐ Rarely ☐ Never
	15	Have you ever referred a patient to a fertility preservation center?	☐ Yes ☐ No
	16	If not, why (multiple answers)?	☐ Lack of information about available centers ☐ Time constraints/therapeutic urgency ☐ High cost for the patient ☐ Other: _____
	17	Are you in favor of implementing a national protocol for fertility preservation in Morocco?	☐ Yes ☐ No
	18	Have you successfully preserved fertility in a patient via gamete cryopreservation?	☐ Yes ☐ No
	19	If yes, approximately for how many patients?	☐ 1-5 ☐ 6-10 ☐ >10
V. Needs and Perspectives	20	Are you interested in (multiple answers):	☐ Specific training on fertility preservation ☐ Collaborations with reproductive centers ☐ Standardized protocols in your department
	21	What priority actions should be implemented in Morocco to improve access to fertility preservation?	
